# Syndrome d’apnées hypopnées obstructives du sommeil du sujet âgé

**DOI:** 10.11604/pamj.2023.44.78.36607

**Published:** 2023-02-07

**Authors:** Imen Sahnoun, Ines Moussa, Rim Ghériani, Racha Smaoui, Leila Douik El Gharbi

**Affiliations:** 1 Service de Pneumologie D, Université de Tunis El Manar, Faculté de Médecine de Tunis, Tunis, Tunisie,; 2 Université de Tunis El Manar, Faculté de Médecine de Tunis, Tunis, Tunisie

**Keywords:** Syndrome d’apnées hypopnées obstructives du sommeil, sujet âgé, polygraphie du sommeil, Sleep apnea hypopnea syndrome, aged, sleep monitoring

## Abstract

**Introduction:**

le syndrome d´apnées hypopnées obstructives du sommeil (SAHOS) du sujet âgé est une affection fréquente mais sous diagnostiquée. L´objectif de notre étude était de déterminer les caractéristiques cliniques et polygraphiques du SAHOS chez les sujets âgés en les comparant à des sujets jeunes.

**Méthodes:**

l´étude rétrospective menée au service de pneumologie D de l´hôpital Abderrahmen Mami de l´Ariana, incluant 222 patients ayant un SAHOS répartis en deux groupes: groupe 1 comportant 72 patients âgés entre 18 et 45 ans et groupe 2 comportant 150 patients âgés de 65 ans et plus. Les données cliniques et polygraphiques ont été recueillies.

**Résultats:**

les apnéiques âgés étaient plus de sexe féminin, moins exposés au tabac mais plus exposés à la fumée de biomasse. Le délai moyen de consultation était plus long pour les apnéiques âgés. La fatigue diurne et les troubles de la mémoire étaient plus fréquents chez les sujets âgés. L´asthme, l´hypothyroïdie, le diabète, la dyslipidémie, l´HTA et l´AC/FA étaient plus fréquents chez les apnéiques âgés. Les apnéiques âgés avaient moins de pauses respiratoires et moins d´hypertrophie amygdalienne. Nous n´avons pas trouvé de différence significative entre les deux groupes concernant la sévérité du SAHOS. L´analyse multivariée a montré que les apnéiques âgés étaient plus prédisposés à être de sexe féminin, à avoir plus de trouble de la mémoire et à avoir plus de comorbidités à type d´HTA, d´AC/FA, de diabète et d´hypothyroïdie.

**Conclusion:**

la fréquence des comorbidités impose une exploration du sommeil chez le sujet âgé apnéique que le tableau clinique soit typique ou non.

## Abstract (en)

**Introduction:**

obstructive sleep apnea hypopnea syndrome (OSAHS) is a common but often under diagnosed disease among elderly persons. The aim of our study was to determine the clinical and polygraphic characteristics of OSAHS in elderly by comparing them to younger patients.

**Methods:**

a retrospective study was performed at Pavillon D pneumology at Abderrahmen Mami Hospital in Ariana including 222 patients with OSAHS, divided into two groups: group 1 including 72 patients aged 18 to 45 years and Group 2 including 150 patients aged 65 and over. Clinical and polygraphic data was collected.

**Results:**

elderly patients were more female, less exposed to tobacco but more exposed to biomass smoke. The average consultation time was significantly longer for elderly patients compared to young patients. Diurnal fatigue and memory impairment were more observed in elderly patients. Asthma, hypothyroidism, diabetes, dyslipidemia, hypertension and atrial fibrillation were most commonly observed in elderly patients. Pauses in airflow and tonsillary hypertrophy were less observed in this group. There was no significant difference in OSAHS severity between the 2 groups. Logistic regression analysis showed that elderly apneic patients were more likely to be female, to have more memory impairment and to have more comorbidities with HTA, atrial fibrillation, diabetes and hypothyroidism.

**Conclusion:**

the frequency of cardiovascular, metabolic and cognitive comorbidities requires sleep investigation in apneic elderly subject, whether the clinical presentation is typical or not.

## Introduction

Le syndrome d´apnées hypopnées obstructives du sommeil (SAHOS) est une pathologie chronique caractérisée par la survenue répétée d´épisodes d´obstruction totale (apnée) ou partielle (hypopnée) des voies aériennes supérieures (VAS) pendant le sommeil [1]. Ces événements respiratoires nocturnes entraînent une fragmentation du sommeil, des hypoxémies nocturnes et des dysfonctionnements diurnes [2]. La prévalence de SAHOS est de 4% chez les hommes et de 2% chez les femmes entre 30 et 60 ans [1]. Cependant, il peut affecter tous les âges et sa prévalence augmente après l´âge de 60 ans pour devenir équivalente entre les deux sexes [3]. La prévalence du SAHOS chez les patients âgés de plus de 65 ans est variable d´une étude à l´autre, entre 30% et 80% [4]. Malgré sa haute prévalence chez le sujet âgé, le SAHOS semble être sous diagnostiqué et sous-traité en pratique gériatrique, pourtant ses conséquences sont nombreuses et particulièrement néfastes dans cette population. La mortalité et la morbidité du SAHOS chez les sujets de plus de 70 ans sont importantes [5]. Les patients ayant un index d´apnées hypopnées (IAH) supérieur à 30 ont ainsi 2,5 fois plus de risque de faire un accident vasculaire cérébral (AVC) ischémique [6]. Il existe par ailleurs des complications cardiaques [7] et neurocomportementales avec un déclin des fonctions cognitives [8]. Plusieurs facteurs peuvent être à l´origine de la méconnaissance du SAHOS, avec en particulier, sa présentation atypique chez le sujet âgé. Cette atypie clinique s´explique d´une part par les changements physiologiques du sommeil liés à l´âge et par la fréquence des comorbidités d´autre part. En effet, les AVC [6], les troubles cognitifs [8], les chutes [9], le glaucoma [10] et l´altération de la qualité de vie [11], bien que non spécifiques, sont souvent associés au SAHOS du sujet âgé. L´objectif principal de notre étude était de déterminer les caractéristiques cliniques et polygraphiques du SAHOS chez les sujets âgés en les comparants à celle des sujets jeunes.

## Méthodes

**Type de l´étude**: il s´agit d´une étude rétrospective, monocentrique, analytique descriptive et comparative, portant sur les dossiers de patients ayant un SAHOS colligés au service de pneumologie D du centre Hospitalo-Universitaire Abderrahmane Mami de l´Ariana, Tunisie durant une période allant de Janvier 2015 jusqu´à Décembre 2018.

### Population cible

**Critères d´inclusion**: les patients inclus dans l´étude étaient des malades âgés entre 18 et 45 ans et de 65 ans et plus ayant un SAHOS défini selon recommandations l'Académie américaine de médecine du sommeil (AASM) [12].

**Critères de non inclusion**: n´ont pas été inclus dans cette étude les patients ayant un syndrome d´apnées centrales du sommeil, les patients présentant un autre trouble non respiratoire du sommeil (narcolepsie, syndrome des jambes sans repos, hypersomnie) ou une pathologie neurologique ou musculaire (myopathie, encéphalopathie…).

### Paramètres étudiés

**Paramètres cliniques**: les patients ont été évalués sur le plan démographique (âge, sexe, tabagisme, exposition à la fumée de bois, indice de masse corporelle (IMC)). Les comorbidités respiratoires, cardiovasculaires et métaboliques associés au SAHOS ont été recherchés. L´obésité était définie par un indice de masse corporelle (IMC) supérieure ou égale 30 kg/m^2^. Les signes cliniques recherchés étaient: ronflement sévère et quotidien, les pauses respiratoires constatées par l´entourage, éveils répétés pendant le sommeil, fatigue diurne, troubles de l´humeur (nervosité), troubles de la sexualité (baisse de la libido, impuissance), céphalées matinales, difficultés de concentration, trouble de la mémoire, nycturie (plus d´une miction par nuit) et somnolence diurne excessive (SDE) évaluée par le score d´Epworth [13]. Il s´agit d´un auto questionnaire qui évalue de 0 (aucun) à 3 (risque important) le risque de somnoler dans huit situations, principalement passives, de la vie quotidienne. Le score s´étend de 0 à 24. Un total de 10 points et plus suggère une somnolence excessive [13].

**Paramètres polygraphiques**: tous nos patients ont bénéficié d´une polygraphie de ventilation pendant une nuit d´hospitalisation. Le polygraphe utilisé était de type Embletta ou Nox T3. Le système relevait six signaux (flux nasal mesuré par le capteur correspondant à une lunette nasale, mouvements thoraciques et abdominaux (RIP) pour relever les efforts respiratoires, oxymétrie pour relever les désaturations nocturnes, fréquence cardiaque, position corporelle et activité). Le scorage des évènements a été réalisé selon les dernières recommandations de l'Académie américaine de médecine du sommeil (AASM). Une apnée obstructive est définie par une chute du signal du flux aérien? Quatre vingt dix pour cent (90%) par rapport à la ligne de base précédant l´événement et ce pendant au moins 10 s avec persistance des efforts ventilatoires pendant l´apnée. Une hypopnée est définie par une chute d'un signal de flux validé d'au moins 30% par rapport au niveau de base pendant au moins 10 secondes associée à une désaturation en oxygène d´au moins 3% et/ou un micro-éveil [12,14]. Le niveau de sévérité du SAHOS est déterminé par la valeur de l´index d´apnées hypopnées du sommeil par heure définissant ainsi trois groupes de sévérité polygraphiques: SAHOS léger avec un IAH entre 5 à 15 par heure, le SAHOS modéré avec un IAH entre 15 à 30 par heure et le SAHOS sévère avec IAH supérieur ou égal à 30 par heure [15]. Nous avons étudié l´index d´apnées hypopnées par heure (IAH), l´index de désaturation par heure (IDO) et la saturation artérielle en oxygène (SaO_2_) moyenne et minimale au cours du sommeil chez nos patients apnéiques.

**Répartition de la population**: les patients ont été répartis en deux groupes: Groupe 1 comportant des patients âgés entre 18 ans et 45 ans; et Groupe 2 comportant des patients âgés de 65 ans et plus. Nous avons comparé les différents paramètres cliniques et polygraphiques entre les deux groupes.

**Analyse statistique**: les données ont été saisies et analysées grâce au logiciel SPSS version 20 (Statistical Package for the Social Sciences). Nous avons calculé les fréquences pour les variables qualitatives. Nous avons aussi calculé les moyennes, les médianes, l´écart type (ET) pour les variables quantitatives. Nous avons utilisé le test de Khi2 ou le test de Fisher pour comparer les variables qualitatives et le test de Student pour comparer les variables quantitatives. Le seuil de signification a été fixé à 5%. Nous avons réalisé ensuite une régression linéaire multiple.

## Résultats

En tout 222 patients ayant un SAHOS confirmé par une polygraphie ventilatoire ont été retenus pour cette étude. Ces patients ont été répartis en deux groupes: groupe 1 comportant 72 patients âgés entre 18 ans et 45 ans et groupe 2 comportant 150 patients âgés de 65 ans et plus. L´âge moyen des patients âgés était de 71.77 ± 6.08 ans. La majorité de nos patients âgés (46.6%) appartiennent à une tranche d´âge située entre 65 et 69 ans. Comparés aux apnéiques jeunes, les apnéiques âgés étaient plus de sexe féminin, moins exposés au tabac mais plus exposés à la fumée de biomasse. L´IMC moyen était significativement moins élevé chez les sujets âgés. La fréquence de l´obésité était similaire entre les apnéiques jeune (87.5%) comparativement aux apnéiques âgés (80%). Le délai moyen de consultation était significativement plus long pour les apnéiques âgés par rapport aux apnéiques jeunes (Tableau 1). Les comorbidités étaient plus observées chez les apnéiques âgés et étaient essentiellement l´asthme, l´hypothyroïdie, le diabète, la dyslipidémie et les comorbidités cardio-vasculaires (88% vs 11%; p<0.001). Ces dernières étaient dominées par l´hypertension artérielle (HTA) et l´arythmie cardiaque par fibrillation auriculaire (AC/FA). L´insuffisance cardiaque et la coronaropathie étaient uniquement notées dans le groupe âgé. Il n´y avait pas de différence significative quant à l'AVC et l´artérite oblitérante des membres inférieurs entre les deux groupes. Nous avons noté trois cas de dépression, tous étaient du groupe des apnéiques âgés. L´hypertrophie amygdalienne était moins observée dans le groupe des apnéiques âgés (Tableau 2).

**Tableau 1:** caractéristiques démographiques selon le groupe d´âge

	[18-45 ans](n=72)	≥ 65 ans(n=150)	p
**Age moyen (ans)**	37.82±6.4	71.77±6.08	<0.001**
**Sexe féminin (n, %)**	31(43.1%)	111 (74%)	<0.001*
**Tabac (n, %)**	23(31.9%)	27(18%)	0.02*
**Exposition à la fumée de bois (n, %)**	2(2.8%)	16(10.7%)	0.04*
**IMC moyen (Kg/m^2^)**	39.81±9.06	36.68±7.21	0.005**
**Délai moyen de consultation (mois)**	39.81	56.98	0.04**

IMC: indice de masse corporelle *Test de Khi2, p<0.05, **Test de Student, p<0.05,

**Tableau 2:** comorbidités selon le groupe d´âge

Variables	[18-45 ans] (n=72)	≥ 65 ans(n=150)	P
**HTA (n, %)**	11(15.3%)	123(82%)	0.001
**AC/FA (n, %)**	2(2.8%)	27(17.3%)	0.001
**Coronaropathie (n, %)**	0	34(22.7%)	<0.001
**Insuffisance cardiaque (n, %)**	0	9(6%)	0.033
**AVC (n, %)**	0	6(4%)	0.18
**AOMI (n, %)**	0	7(4.7%)	0.09
**Asthme(n, %)**	1(1.4%)	19(12.7%)	0.005
**BPCO (n, %)**	0	8(5.3%)	0.27
**Syndrome obésité hypoventilation (n, %)**	1(1.4%)	2(1.3%)	1
**Diabète (n,%)**	14(19.4%)	88(58.7%)	<0.001
**Dyslipidémie (n, %)**	26(36.1%)	88(58.7%)	0.002
**Hypothyroïdie (n, %)**	2(2.8%)	20(13.5%)	0.01
**Hypertrophie amygdalienne (n, %)**	7(9.7%)	2 (1.3%)	0.006

HTA : hypertension artérielle, AC/FA : arythmie cardiaque par fibrillation auriculaire, AVC : accident vasculaire cérébrale, AOMI : artérite oblitérante du membre inférieur, BPCO: bronchopneumopathie chronique obstructive Test de Khi2, p<0.05

De point de vue clinique, aucune différence significative n´a été constaté entre les deux groupes en termes de ronflement nocturne, de somnolence diurne excessive, de nycturie et de difficultés de concentration. Le score d´Epworth moyen était similaire dans les deux groupes (7.11 ± 5.97 vs 7.63 ± 6.1; p=0.601). Cependant, la fatigue diurne et les troubles de la mémoire étaient significativement plus fréquents chez les sujets âgés. Les pauses respiratoires étaient moins rapportées par l´entourage dans ce groupe (Tableau 3). Il n´y avait pas de différence entre les deux groupes en termes de sévérité du SAHOS. L´IAH moyen, l´IDO moyen, la saturation moyenne au cours du sommeil et la saturation minimale moyenne au cours du sommeil étaient similaires entre les deux groupes (Tableau 4). De l´analyse multivariée, il ressort que les apnéiques âgés étaient plus prédisposés à être de sexe féminin (p=0.03, OR= 2.79, IC95%: 1.07-7.30), à avoir des troubles de la mémoire (p=0.005, OR= 14.10, IC95%: 2.26-88.02) et à avoir des comorbidités à type d´HTA (p<0.001, OR= 52.65, IC95%: 16.72-165.80), d´AC/FA (p=0.017, OR= 11.86, IC95%: 1.56-89.68), de diabète (p=0.03, OR= 2.81, IC95%: 1.07-7.36) et d´hypothyroïdie (p=0.03, OR= 8.037, IC95%: 1.14-56.25).

**Tableau 3:** symptômes nocturnes et diurnes selon le groupe d’âge

Variables	[18-45 ans](n=72)	≥ 65 ans(n=150)	p
**Somnolence diurne excessive (n, %)**	29(40.3%)	75(50%)	0.174
**Fatigue diurne (n, %)**	16 (22.2%)	56 (37.7%)	0.02
**Céphalées matinales (n, %)**	18(25%)	31(20.7%)	0.466
**Troubles de la sexualité (n, %)**	3(4.2%)	1(0.7%)	0.101
**Troubles de l´humeur (n, %)**	7(9.7%)	8(5.3%)	0.25
**Troubles de la mémoire (n, %)**	3(4.2%)	19(12.7%)	0.04
**Troubles de la concentration (n, %)**	7(9.7%)	21(14%)	0.36
**Pauses respiratoires au cours du sommeil (n, %)**	37 (51.4%)	54(36%)	0.02
**Ronflements (n, %)**	63 (87.5%)	124(82.7%)	0.35
**Eveils répétés (n, %)**	17(23.6%)	40(26.7%)	0.62
**Nycturie (n, %)**	9(12.5%)	28(18.7%)	0,24

Test de Khi2, p<0.05

**Tableau 4:** données polygraphiques selon le groupe d´âge

Variables	[18-45 ans] (n=72)	≥ 65 ans(n=150)	p
**Différents SAHOS**			
**SAHOS léger**	21(29.2%)	29 (19.3%)	0.23*
**SAHOS modéré**	9(12.5%)	25(16.7%)	
**SAHOS sévère**	42(58.3%)	96(64%)	
**IAH moyen (/h)**	34.56±25.65	33.64±19.61	0.78**
**IDO moyen (/h)**	34.49±25.65	33.20±20.40	0.78**
**SaO_2_ moyenne au cours du sommeil(%)**	91.98±3.96	91.25±2.95	0.17**
**SaO_2_ minimale au cours du sommeil (%)**	74.69±14.09	75.22±9.4	0.76**

IAH : Index d´apnées-hypopnées, IDO: Index de désaturation, SaO_2_: Saturation en oxygène *Test de Khi2, p<0.05, **Test de Student, p<0.05

## Discussion

Le SAHOS est une affection hautement prévalente chez le sujet âgé. Plusieurs études ont mis en évidence une augmentation de sa prévalence avec l´âge [4,16]. Dans notre étude, la majorité de nos patients âgés (46.6%) appartiennent à une tranche d´âge située entre 65 et 69 ans. Cette augmentation de la prévalence du SAHOS avec l´âge peut être expliquée par des modifications liées au vieillissement physiologiques [17] mais aussi du fait des comorbidités fréquentes dans cette tranche d´âge. Chez l´adulte, le SAHOS est plus fréquent chez l´homme que chez la femme [1,18] du fait du rôle protecteur des hormones féminines [19]. Cette différence s´atténue après la ménopause [1,19] ce qui explique que le SAHOS chez le sujet âgé, atteint de façon presque équivalente les 2 sexes [3,4]. Nous avons trouvé une prédominance masculine dans le groupe des sujets jeunes (56.9% vs 26%) dans notre étude. Cependant nous avons noté une prédominance féminine (74% vs 43.1%) dans le groupe des apnéiques âgés comparativement à celui des apnéiques jeunes.

Concernant les habitudes de vie, il a été démontré que la prévalence de SAHOS est augmentée chez les fumeurs. Le tabac favorise la survenue des événements respiratoires au cours du sommeil par l´inflammation chronique des VAS [20] mais aussi par la privation de l´effet stimulant de la nicotine au cours du sommeil [21]. Dans notre étude, le nombre de fumeur était moins important dans le groupe des apnéiques âgés. Par ailleurs, ce groupe de patients étaient plus exposés à la fumée de biomasse. En effet la fumée de biomasse semble avoir un effet semblable à celui du tabac sur les VAS. Concernant la prise de médicaments hypnotiques, fréquente chez les sujets âgés et favorisant l´apparition d´apnées par l´aggravation de l´hypotonie des muscles dilatateurs des VAS au cours du sommeil, celle-ci n´a été retrouvée que chez trois patients âgés souffrant d´une dépression. Contrairement à l´adulte jeune, la surcharge pondérale n´est pas aussi fréquente chez le sujet âgé apnéique [17,22,23]. Cette constatation clinique sous-entend une physiopathologie différente de celle de l´adulte jeune. En effet, le vieillissement prédispose au collapsus des VAS et entraîne des modifications d´ordre mécanique et neurologique. La diminution du diamètre, de la tonicité musculaire, l´augmentation des résistances des VAS [24] et l´édentation [25] peuvent jouer un rôle important dans l´augmentation de la compliance des VAS. L´instabilité du contrôle ventilatoire secondaire aux éveils fréquents [26], la diminution du reflexe des VAS et de la réponse ventilatoire à l'hypoxie [27] contribuent à la genèse du SAHOS chez les sujets âgés. Dans notre étude, La fréquence de l´obésité était similaire entre les deux groupes. L´IMC moyen était significativement moins élevé chez les sujets âgés.

Parfois, le SAHOS coexiste, sans être diagnostiqué, chez les patients âgés présentant une maladie cardiovasculaire. Les résultats obtenus à partir de l´inclusion de 6132 sujets dans la « Sleep heart health study » [28], dont près de la moitié étaient des sujets de plus de 65 ans, ont montré le rôle prédictif des troubles respiratoires nocturnes dans la survenue d'une HTA. Il a été démontré aussi que la maladie coronaire a été associée de manière indépendante au SAHOS avec un risque relatif de 1,27 [29]. Ainsi le SAHOS aide à identifier les patients à haut risque de maladies coronariennes [30]. La prévalence des apnées chez des patients porteurs de troubles du rythme et de la conduction est élevée. Garrigue *et al*. [31] ont rapporté que près des 2/3 des patients porteurs de pacemaker (d´âge moyen 64 ans) pour une insuffisance cardiaque, une bradycardie ou un bloc auriculo-ventriculaire, sont porteurs d´un SAHOS. Gami *et al*. [32] ont retrouvé une prévalence de SAHOS de 49% chez les patients âgés en fibrillation auriculaire contre 32% dans la population cardiologique âgée témoin.

De même, la présence d´un SAHOS chez les sujets âgés était associée à une majoration du risque d´IC (Odds ratio=2,38) indépendamment des autres facteurs de risque connus [29]. Chez le sujet âgé de plus de 70 ans, l´existence d´un SAHOS sévère multiplie par 2.5 le risque d´AVC ischémique, indépendamment des autres facteurs de risque y compris l´HTA [6]. Ainsi, le SAHOS du sujet âgé pourrait représenter un facteur de risque supplémentaire. Le pronostic vital à long terme des apnéiques âgés est essentiellement lié à la survenue d´événements cardio-vasculaires. Martínez-García *et al*. [7] ont rapporté qu´un SAHOS sévère non traité par une ventilation par Pression Positive Continue (PPC) chez des sujets âgés, est associé à deux fois plus de mortalité cardiovasculaire par AVC ou par IC, que ceux sans SAHOS. Les résultats de notre étude rejoignent les données de la littérature. Les comorbidités cardio-vasculaires étaient plus observées chez les apnéiques âgés. Ces dernières étaient dominées par l´HTA et la FA. L´insuffisance cardiaque et la coronaropathie étaient uniquement notées dans le groupe âgé. En analyse multivariée, les apnéiques âgés de plus de 65 ans étaient plus prédisposés à avoir de l´HTA et de la FA. La relation entre le SAHOS du sujet âgé et le diabète est bidirectionnelle: d´une part, du fait de vieillissement, le SAHOS accélère la progression vers l´intolérance au glucose, par le biais de l´activation du système nerveux sympathique et du stress oxydatif. D´autre part, le diabète type 2 potentialise l´effet du vieillissement sur les VAS et la musculature [33]. Zhuo *et al*. [34] ont démontré que les patients âgés hypertendus avec un SAHOS présentaient des niveaux significativement élevés de glycémie à jeun, de glycémie postprandiale, d'hémoglobine glyquée, de cholestérol total et de TG par rapport au groupe âgé sans SAHOS. Dans notre étude, les sujets âgés apnéiques avaient significativement plus de diabète, de dyslipidémie et d´hypothyroïdie comparativement aux apnéiques jeunes. En analyse multivariée, les apnéiques âgés ont tendance à avoir plus de diabète et d´hypothyroïdie.

Quant aux comorbidités respiratoires, l´association d´une BPCO et d´un SAHOS chez le sujet âgé est le fait du hasard. Toute fois l´asthme est souvent associé au SAHOS [35]. Dans notre étude, la BPCO était notée uniquement chez huit apnéiques âgés. Cependant l´asthme était significativement plus marqué dans le groupe des sujets âgés comparativement à celui des sujets jeunes. Ceci pourrait être expliqué par la prédominance féminine dans ce groupe. Si la somnolence diurne excessive est le principal des symptômes chez l´adulte d´âge moyen, elle est peu évoquée comme une plainte principale chez le sujet âgé [36]. Elle peut se limiter à une sensation de fatigue plus ou moins permanente et plus ou moins intense comme elle peut se manifester par des endormissements intempestifs [37]. Elle est rarement ressentie: seulement 25% des hommes ≥ 60 ans ayant un SAHOS sévère ont un score d´Epworth pathologique [38]. Le plus souvent c´est la constatation par l´entourage. L´Observation and interview based diurnal sleepiness inventory (ONSI) est un questionnaire conçu pour les patients gériatriques en institution, remplie par le personnel médical et comporte trois questions sur les endormissements et leur durée estimée sur la journée. Il est validé en France chez les patients âgés ayant un SAHOS avec une sensibilité de 84% et une spécificité de 85% [39]. Par ailleurs, les chutes fracturaires liées à la SDE sont fréquentes chez les apnéiques âgés [9]. Dans notre étude, 40,3% des sujets jeunes et 50% des sujets âgés présentaient une SDE sans différence significative entre les deux groupes. Nous n´avons pas noté de chutes fracturaires secondaires à la SDE dans notre étude. Cependant, la fatigue diurne était plus fréquente dans le groupe des sujets âgés apnéiques.

Une faible prévalence du ronflement chez les apnéiques âgés est rapportée dans la littérature [40]. Endeshaw a montré l´absence de corrélation significative entre le ronflement, les pauses respiratoires et un IAH > 15 chez le sujet âgé [41]. Dans notre étude, la fréquence du ronflement été élevée et similaires dans les deux groupes. D´autres manifestations du SAHOS peuvent être au premier plan chez le sujet âgé tel que les pauses respiratoires, les réveils brusques nocturnes, les céphalées matinales et la nycturie [42]. Dans notre étude, les pauses respiratoires étaient plus fréquentes chez les apnéiques jeunes que les apnéiques âgés. Nous n´avons pas trouvé de différence significative entre les deux groupes concernant les réveils brusques, les céphalées matinales, la nycturie et les troubles sexuels. Les pauses respiratoires étaient plus fréquentes chez les apnéiques jeunes que les apnéiques âgés. Le SAHOS serait associé à des troubles de l´attention, de la vigilance, de la performance psychomotrice, de la mémoire et des fonctions exécutives [8,43]. Le SAHOS chez le sujet âgé est associé à un risque plus élevé de *Mild Cognitive Impairment* (*MCI*) et ce en rapport avec la durée des apnées et des hypopnées [44]. Ceci a été expliqué par une charge amyloïde plus importante secondaire à la sévérité des épisodes hypoxiques, ainsi qu´une augmentation du volume de la substance grise, du métabolisme et de la perfusion cérébrale principalement dans le cortex cingulaire postérieur et le précunéus chez les sujets âgés apnéiques [45]. Deux études ont comparé les fonctions cognitives entre les sujets témoins et les sujets avec SAHOS (IAH>15/h), d´âge moyen de 68 ans, non déments. Les résultats suggèrent que chez les sujets âgés sans plainte cognitive, les troubles cognitifs seraient présents à minima et seulement dans les SAHOS sévères (IAH> =30/h) [46,47]. D´autres études ont montré que le lien entre le déclin cognitif et le SAHOS ne serait pas expliqué par la sévérité en termes d´IAH, mais plutôt par l´importance et la profondeur de l´hypoxie [48].

Dans notre échantillon, les troubles de la mémoire ont intéressé plus les sujets âgés. Les troubles de la concentration, de la sexualité et de l´humeur étaient similaires entre les deux groupes. En analyse multivariée, il s´avère que les apnéiques âgés de plus de 65 ans étaient plus prédisposés à avoir plus de trouble de mémoire que les sujets jeunes. La polygraphique ventilatoire du sommeil est une alternative à la polysomnographie chez le sujet âgé. Dans une large étude européenne, Deegan a montré que le nombre d´apnées-hypopnées augmente avec l´âge [49]. Bearpark H n´a pas trouvé de corrélation entre l´âge et les troubles respiratoires au cours du sommeil mais l´augmentation de l´âge semble être associée à la saturation minimale au cours du sommeil (SaO_2_< 85%) [50]. Dans notre étude, l´IAH moyen, l´index de désaturation moyen, la saturation moyenne au cours du sommeil et la saturation minimale moyenne au cours du sommeil étaient similaires entre les deux groupes. Le SAHOS sévère était le plus fréquent dans les deux groupes sans différence significative.

Notre étude a ciblé une population particulière, la population gériatrique, afin d´attirer l´attention des médecins de première ligne ainsi que les gériatres quant à la particularité du SAHOS chez le sujet âgé. La particularité du SAHOS du sujet âgé dans notre étude était son apparition chez des patients porteurs de comorbidités fréquentes liées à l´âge mais pouvant s´aggraver sous l´effet des troubles respiratoires nocturnes et en masquer les symptômes nécessaires au diagnostic positif. La présentation atypique du SAHOS chez le sujet âgé, dont la présence des troubles cognitifs pouvant être prédictifs de SAHOS dans cette population. Cependant, ce travail n'est pas sans limites. D'abord, il s´agit d´un travail rétrospectif, donc on ne peut pas maitriser toutes les données cliniques et para cliniques, ce qui peut être une insuffisance dans le recueil des données et de leurs interprétations ultérieures. En effet, nous n´avons pas pu évaluer de façon objective (par des scores) le degré des troubles cognitifs de nos malades. L´idéal serait de réaliser des études prospectives prolongées prenant en compte une population large.

## Conclusion

Le syndrome d´apnées hypopnées obstructives du sommeil du sujet âgé est une entité particulière dont la présentation clinique était dominée par la fatigue diurne et les troubles de la mémoire dans notre étude. Cette présentation clinique pourrait être masquée par les comorbidités, rend difficile le diagnostic et par conséquent retarde la prise en charge du SAHOS dans cette population gériatrique. Les comorbidités cardiovasculaires et métaboliques pourraient être expliquées par l´âge avancé mais aussi par le retentissement du SAHOS lui-même. Ces données suggèrent qu´un SAHOS chez le sujet âgé doit être recherché devant une détérioration cognitive d´un patient âgé, mais aussi devant un déséquilibre de sa pathologie cardiovasculaire ou métabolique.

### Etat des connaissances sur le sujet

- *Il existe une haute prévalence du SAHOS chez le sujet âgé;*
- *La mortalité et la morbidité du SAHOS chez les sujets âgés sont importantes*.

### Contribution de notre étude à la connaissance

- *Notre travail montre que la présentation clinique typique du SAHOS chez le sujet âgé est atypique l´origine de la méconnaissance du SAHOS;*
- *Cette atypie clinique s´explique d´une part par les changements physiologiques du sommeil liés à l´âge et par la fréquence des comorbidités d´autre part*.
